# Association of Intramuscular Fat Infiltration With Incident Venous Thromboembolism: A Population‐Based Cohort Study

**DOI:** 10.1002/jcsm.70342

**Published:** 2026-07-09

**Authors:** Ning Wang, Yuqing Zhang, Weiya Zhang, Michael Doherty, Nancy E. Lane, Jie Wei, Changjun Li, Guanghua Lei, Chao Zeng, Yilun Wang

**Affiliations:** ^1^ Department of Orthopaedics Xiangya Hospital, Central South University Changsha China; ^2^ Division of Rheumatology, Allergy, and Immunology, Department of Medicine Massachusetts General Hospital, Harvard Medical School Boston Massachusetts USA; ^3^ Academic Rheumatology, School of Medicine University of Nottingham Nottingham UK; ^4^ Pain Centre Versus Arthritis UK Nottingham UK; ^5^ Center for Musculoskeletal Health and Department of Medicine University of California School of Medicine Sacramento California USA; ^6^ Hunan Key Laboratory of Joint Degeneration and Injury Changsha China; ^7^ Key Laboratory of Aging‐Related Bone and Joint Diseases Prevention and Treatment, Ministry of Education Xiangya Hospital, Central South University Changsha China; ^8^ National Clinical Research Center for Geriatric Diseases (Xiangya Hospital of Central South University) Changsha China; ^9^ Department of Epidemiology and Health Statistics, Xiangya School of Public Health Central South University Changsha China; ^10^ FuRong Laboratory Changsha China; ^11^ Department of Endocrinology, Endocrinology Research Center Xiangya Hospital, Central South University Changsha China

**Keywords:** deep vein thrombosis, intramuscular fat infiltration, pulmonary embolism, venous thromboembolism

## Abstract

**Background:**

Venous thromboembolism (VTE) is a major acute cardiovascular condition with high mortality, affecting nearly 10 million individuals worldwide each year. Identifying novel and modifiable risk factors is crucial for advancing prevention strategies. Intramuscular fat infiltration (IMFI), a modifiable condition linked to inflammation and muscle weakness, both established contributors to VTE risk, has not been previously studied in relation to incident VTE. We aimed to examine the association between thigh IMFI and incident VTE, including pulmonary embolism (PE) and deep vein thrombosis (DVT).

**Methods:**

This population‐based cohort study included 24 529 UK Biobank participants with baseline IMFI assessed using magnetic resonance imaging of the thigh muscles. The primary outcome was incident VTE. Secondary outcomes included incident PE and DVT. Cox proportional hazards models estimated hazard ratios (HRs) and 95% confidence intervals (CIs) for the associations of IMFI with incident VTE, PE and DVT, adjusting for potential confounders. Dose–response relationships were evaluated using restricted cubic spline regression models.

**Results:**

Over a mean follow‐up of 4.92 years, 227 participants developed incident VTE. The incidence rates across increasing age‐ and sex‐specific anterior thigh IMFI quartiles were 1.34, 1.28, 1.58 and 3.34 per 1000 person‐years, respectively. Compared with the lowest anterior thigh IMFI quartile, adjusted HRs for incident VTE were 0.88 (95% CI: 0.57, 1.37), 1.02 (95% CI: 0.67, 1.55) and 1.88 (95% CI: 1.26, 2.80) for the second, third and fourth quartiles, respectively. Similar associations were observed for incident PE and DVT. Restricted spline regression models revealed that VTE risk increased progressively across higher IMFI levels. PE and DVT risk showed similar patterns. Analyses using posterior thigh IMFI showed generally consistent associations with incident VTE, PE and DVT.

**Conclusions:**

Elevated thigh IMFI was associated with higher risks of VTE, PE and DVT. These findings identify thigh IMFI as a potential modifiable risk factor for VTE and support further investigation of strategies targeting muscle fat infiltration for thrombotic disease prevention.

**Abbreviations**
BMI: body mass index
CCI: Charlson comorbidity index
CI: confidence interval
DVT: deep vein thrombosis
HMB: β‐hydroxy β‐methylbutyrate
HR: hazard ratio
ICD: International Classification of Diseases
IMFI: intramuscular fat infiltration
IQR: interquartile range
MET: metabolic equivalent
MRI: magnetic resonance imaging
PE: pulmonary embolism
SD: standard deviation
VTE: venous thromboembolism

## 1 Introduction

Venous thromboembolism (VTE), which includes pulmonary embolism (PE) and deep vein thrombosis (DVT), is the third most common acute cardiovascular syndrome globally [1]. With a lifetime incidence of approximately 8%, VTE affects nearly 10 million individuals worldwide each year and is associated with substantial morbidity and mortality [2, 3]. The burden of VTE extends beyond the acute event, encompassing complications from anticoagulant therapy, such as major bleeding, as well as long‐term disability [2, 3]. Despite extensive research, the full spectrum of VTE risk factors remains incompletely understood, limiting advances in risk stratification and prevention. Identifying novel and modifiable risk factors is therefore critical for improving preventive strategies.

Intramuscular fat infiltration (IMFI), characterized by adipose tissue deposition within skeletal muscle, increases by 6%–8% annually in aging populations [4, 5]. IMFI has been linked to elevated levels of proinflammatory cytokines, chemokines and adipokines [6], which promote localized myosteatosis and systemic inflammation, both potential contributors to VTE pathogenesis [6, 7]. Furthermore, IMFI is associated with declining skeletal muscle function [8], which plays a pivotal role in venous return and prevention of venous stasis, key components in VTE prevention [9]. Despite these plausible mechanistic pathways, the association between IMFI and VTE risk has not been directly investigated. Notably, IMFI is considered a modifiable factor, making it a promising target for intervention. For example, a randomized controlled trial demonstrated that omega‐3 fatty acid supplementation may prevent IMFI progression [10], while nutritional interventions, such as supplementation with β‐hydroxy β‐methylbutyrate (HMB), have been associated with IMFI reduction [11]. Clarifying the relationship between IMFI and VTE risk could thus open new avenues for mitigating VTE incidence.

To address this knowledge gap, we used data from the UK Biobank to examine the association between thigh IMFI and incident VTE, including separate analyses for PE and DVT. We also assessed the dose–response relationship using restricted cubic spline modelling.

## 2 Methods

### 2.1 Data Source and Study Population

The UK Biobank is a large prospective cohort study that recruited over 500 000 participants from the UK National Health Service between 2006 and 2010 (Research Ethics Committee reference 11/NW/0382). This study was conducted under UK Biobank Application Number 77646. All participants provided written informed consent. Baseline data collection included questionnaires, physical examinations and linkage to national health records for longitudinal follow‐up. Beginning in 2014, a subgroup of 100 000 participants was invited to participate in a multimodal imaging study, which included magnetic resonance imaging (MRI) of skeletal muscle. For the primary analysis, we included individuals with available IMFI assessments in at least one anterior thigh muscle (*n* = 24 847), using the imaging date as the index (baseline) date. Participants with a history of VTE before the index date or missing IMFI data were excluded from the primary analysis. Similar exclusion criteria were applied in the separate analyses of PE and DVT.

### 2.2 Intramuscular Fat Infiltration Measurements

MRI scans were performed using a Siemens MAGNETOM Aera 1.5‐T scanner (Siemens Healthineers, Erlangen, Germany) with a 6‐min dual‐echo Dixon Vibe protocol to generate fat‐ and water‐separated volumetric images from the neck to the knees [12]. Body composition was analysed using AMRA Researcher software (AMRA Medical AB, Linköping, Sweden), which has demonstrated excellent reproducibility compared with manual segmentation, with intraclass correlation coefficients ranging from 0.98 to 1.00 [12, 13]. IMFI was quantified as the average fat fraction within the viable muscle tissue of the right and left thighs, excluding fat‐free muscle volume and was analysed as a proportional measure. When data were unavailable for one leg, the measurement from the available leg was used [14]. The UK Biobank imaging protocol followed standardized acquisition procedures across scanners and sites, including harmonized sequence parameters and centralized quality assurance procedures, which have been shown to minimize inter‐scanner and inter‐site variability [12, 15].

### 2.3 Assessment of Outcomes

The primary outcome was incident VTE, with PE and DVT analysed as secondary outcomes. Events were identified using International Classification of Diseases, Ninth and Tenth Revision (ICD‐9 and ICD‐10) codes [16]. Detailed ICD code definitions for VTE, PE and DVT are provided in Table S1. Diagnoses were classified as incident events if they occurred after the index date and no prior history of VTE was recorded in the health data. Previous studies have demonstrated high validity of ICD‐coded venous thromboembolism outcomes [17].

### 2.4 Assessment of Covariates

Covariates were selected based on existing literature and biologically plausible associations with both exposure and outcome [2, 18, 19, 20]. Age was calculated as the difference between date of birth and the index date. Sex and ethnicity were self‐reported. Body mass index (BMI) was calculated as weight in kilograms divided by height in meters squared (kg/m^2^) based on physical measurements. Smoking and alcohol consumption status were self‐reported via touchscreen questionnaire and categorized as never, previous, current or unknown. Educational attainment was classified as higher education, upper secondary, lower secondary, vocational, other or unknown. Physical activity was assessed using the short‐form International Physical Activity Questionnaire and categorized as low (< 600 metabolic equivalent [MET]‐min/week), moderate (600 to < 3000 MET‐min/week), or high (≥ 3000 MET‐min/week). Frailty status was defined using the Fried frailty phenotype adapted for UK Biobank data. The Charlson comorbidity index (CCI) was derived from ICD‐coded diagnoses [21]. Recent surgery was defined based on hospital inpatient records using OPCS‐4 procedure codes. Abnormalities of gait and mobility and fracture were ascertained using ICD‐10 diagnostic codes recorded in hospital inpatient records. Sarcopenia was defined according to the European Working Group on Sarcopenia in Older People 2 criteria, using sex‐specific cut‐off values for appendicular muscle mass index and handgrip strength. Medication use, including anticoagulants, glucocorticoids, aspirin and hormone therapy, was assessed based on self‐reported information. Age, sex, BMI, education, smoking status, alcohol consumption, physical activity level, white ethnicity, frailty status and medication use were assessed at the initial assessment visit (instance 0). Abnormalities of gait and mobility, fracture and CCI were assessed at any time before the index date. Recent surgery was assessed within 1 year before the index date. Sarcopenia was defined using the latest available measurements from UK Biobank assessment instances 0–3 before the index date.

### 2.5 Statistical Analysis

Baseline characteristics were summarized as means with standard deviations (SD) for normally distributed continuous variables, as medians with interquartile ranges (IQR) for skewed continuous variables, and as percentages for binary or categorical variables. Follow‐up began on the index date and continued until the first occurrence of VTE, death, loss to follow‐up or 31 December 2021, whichever came first. Since age and sex were strongly associated with IMFI and VTE risk, participants were stratified by sex and then grouped into two‐year age categories. Within each sex‐ and age‐specific stratum, participants were assigned to IMFI quartiles based on the stratum‐specific distribution. Participants were then pooled across strata according to their assigned age‐ and sex‐specific IMFI quartiles. IMFI quartiles were defined as Q1 (lowest) to Q4 (highest). Incidence rates of VTE were calculated for each IMFI quartile. Cumulative incidence curves were generated using the Aalen‐Johansen method to account for the competing risk of death. Cox proportional hazards models were used to estimate hazard ratios (HRs) and 95% confidence intervals (CIs) for incident VTE across IMFI quartiles, with Q1 serving as the reference group. The proportional hazards assumption was assessed using Schoenfeld residuals. Multivariable models were adjusted for age, sex, BMI, educational attainment, smoking status, alcohol consumption, physical activity levels, white ethnicity, frailty status, CCI, recent surgery, abnormalities of gait and mobility, fracture, sarcopenia and use of anticoagulant, glucocorticoid, aspirin and hormone therapy. The dose–response relationship was assessed using restricted cubic spline regression models with three knots placed at the 10th, 50th and 90th percentiles of the IMFI distribution. The median IMFI value was used as the reference. The spline models were adjusted for the same covariates as the Cox proportional hazards models. Nonlinearity was assessed using Wald tests for the nonlinear spline terms.

As a sensitivity analysis, we performed imputation analyses to account for missing data. Missing values for the variables listed above were imputed using a sequential regression method with a set of covariates as predictors. Five imputed datasets were generated, and effect estimates were obtained from each dataset and combined using Rubin's rules. To assess the robustness of the observed associations to potential unmeasured confounding, we calculated *E* values to quantify the minimum strength of association that an unmeasured confounder would need to have with both IMFI and VTE to fully explain away the observed associations. Additional analyses using posterior thigh IMFI measurements were performed using the same Cox proportional hazards models and covariate adjustments as in the primary analyses. Subgroup analyses stratified by sex, age (< 60 vs. ≥ 60 years), and BMI (< 30 vs. ≥ 30 kg/m^2^) were conducted to examine the associations between IMFI and VTE across different population subgroups. The same analytical framework was applied separately to PE and DVT outcomes.

A two‐sided *p* value of < 0.05 was considered statistically significant. All analyses used SAS V.9.4 (SAS Institute, Cary, NC, USA).

## 3 Results

**FIGURE 1:**
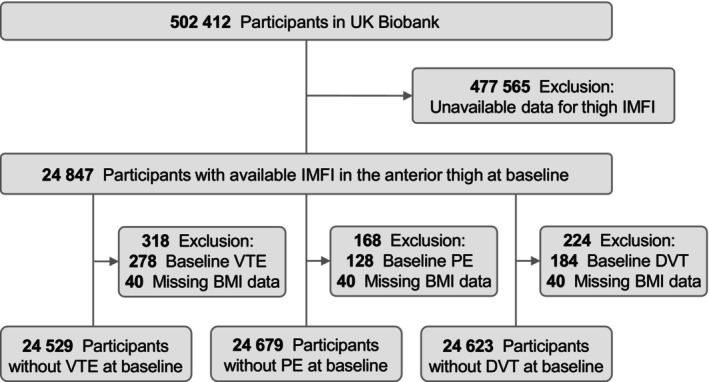
Flow chart. BMI, body mass index; IMFI, intramuscular fat infiltration; VTE, venous thromboembolism.

A flowchart of participant selection is shown in Figure 1. A total of 24 529 participants were included in the analysis of the association between baseline anterior thigh IMFI and incident VTE (Table 1). The median age was 56 years (IQR: 49–61), and 47.9% were male. Compared with those in the lowest age‐ and sex‐specific IMFI quartile (reference group), participants in higher IMFI quartiles tended to have higher BMI, lower educational attainment, a higher prevalence of smoking, lower levels of physical activity, a higher prevalence of frailty, and a greater burden of comorbidities. Baseline characteristics for the PE (*n*: 24 679) and DVT (*n*: 24 623) cohorts across IMFI quartiles were similar to those observed in the VTE cohort (Tables S2 and S3).

**TABLE 1:** Baseline characteristics of participants in VTE analysis.

	Age‐ and sex‐specific quartile of intramuscular fat infiltration of anterior thigh				Standardized mean difference
	Q1 (lowest) (3.17–7.70)	Q2 (4.67–8.65)	Q3 (5.36–10.12)	Q4 (highest) (6.21–27.49)	
Participants, *n*	6118	6138	6144	6129	
Age, years (median [IQR])	56 (49–61)	56 (49–61)	56 (49–61)	56 (49–61)	0.002
Sex (male), *n* (%)	2928 (47.9)	2938 (47.9)	2940 (47.9)	2934 (47.9)	< 0.001
BMI, kg/m^2^ (median [IQR])	24.0 (22.3–26.1)	25.4 (23.4–27.6)	26.6 (24.4–29.1)	28.8 (26.2–32.1)	0.725
Education, *n* (%)					0.205
Higher education	4177 (68.3)	3871 (63.1)	3512 (57.2)	3169 (51.7)	
Upper secondary	406 (6.6)	508 (8.3)	519 (8.4)	513 (8.4)	
Lower secondary	946 (15.5)	1025 (16.7)	1224 (19.9)	1402 (22.9)	
Vocational	225 (3.7)	293 (4.8)	324 (5.3)	331 (5.4)	
Other	273 (4.5)	348 (5.7)	430 (7.0)	574 (9.4)	
Missing	91 (1.5)	93 (1.5)	135 (2.2)	140 (2.3)	
Smoking, *n* (%)					0.173
Current	250 (4.1)	361 (5.9)	421 (6.9)	533 (8.7)	
Previous	1744 (28.5)	1879 (30.6)	2119 (34.5)	2316 (37.8)	
Never	4114 (67.3)	3887 (63.3)	3590 (58.5)	3258 (53.2)	
Missing	10 (0.2)	11 (0.2)	14 (0.2)	22 (0.4)	
Alcohol, *n* (%)					0.028
Current	5834 (95.4)	5835 (95.1)	5834 (95.0)	5813 (94.9)	
Previous	130 (2.1)	135 (2.2)	129 (2.1)	150 (2.4)	
Never	153 (2.5)	166 (2.7)	175 (2.8)	162 (2.6)	
Missing	1 (0.0)	2 (0.0)	6 (0.1)	4 (0.1)	
Physical activity level^a^, *n* (%)					0.181
Low	689 (11.3)	871 (14.2)	1034 (16.8)	1288 (21.0)	
Moderate	2911 (47.6)	2873 (46.8)	2759 (44.9)	2534 (41.3)	
High	1676 (27.4)	1533 (25.0)	1317 (21.4)	1244 (20.3)	
Missing	842 (13.8)	861 (14.0)	1034 (16.8)	1063 (17.3)	
White ethnic, *n* (%)	5954 (97.3)	5955 (97.0)	5941 (96.7)	5951 (97.1)	0.019
Frailty status, *n* (%)					0.243
Robust	4238 (69.3)	3924 (63.9)	3476 (56.6)	3025 (49.4)	
Pre‐frail	1382 (22.6)	1629 (26.5)	1975 (32.1)	2245 (36.6)	
Frail	15 (0.2)	43 (0.7)	48 (0.8)	125 (2.0)	
Missing	483 (7.9)	542 (8.8)	645 (10.5)	734 (12.0)	
Weighted CCI, *n* (%)					0.132
CCI = 0	5296 (86.6)	5122 (83.4)	4979 (81.0)	4737 (77.3)	
CCI > 0	822 (13.4)	1016 (16.6)	1165 (19.0)	1392 (22.7)	
Recent surgery, *n* (%)	620 (10.1)	710 (11.6)	757 (12.3)	876 (14.3)	0.068
Abnormalities of gait and mobility, *n* (%)	3 (0.0)	5 (0.1)	13 (0.2)	15 (0.2)	0.032
Fracture, *n* (%)	106 (1.7)	104 (1.7)	118 (1.9)	136 (2.2)	0.021
Sarcopenia, *n* (%)	43 (0.7)	27 (0.4)	24 (0.4)	15 (0.2)	0.035
Medication, *n* (%)					
Anticoagulant	24 (0.4)	26 (0.4)	39 (0.6)	77 (1.3)	0.053
Glucocorticoid	150 (2.5)	186 (3.0)	195 (3.2)	260 (4.2)	0.051
Aspirin	531 (8.7)	579 (9.4)	692 (11.3)	809 (13.2)	0.083
Hormone therapy	1009 (16.5)	1066 (17.4)	1114 (18.1)	1145 (18.7)	0.032
Death, *n* (%)	68 (1.1)	79 (1.3)	99 (1.6)	124 (2.0)	0.041

*Note:* Charlson comorbidity index (including cancer, cerebrovascular disease, chronic obstructive pulmonary disease, dementia, diabetes, heart failure, myocardial infarction, hemiplegia, AIDS, liver disease, chronic kidney disease, other chronic pulmonary disease, peripheral vascular disease, rheumatic diseases and peptic ulcer disease) was calculated with scores ranging from 0 to 10. For analysis, weighted CCI was dichotomized into two groups: 0 (no comorbidity burden) and > 0 (any comorbidity burden). Age, sex, BMI, education, smoking, alcohol, physical activity level, white ethnicity, frailty status and medication use were assessed at the initial assessment visit (instance 0); abnormalities of gait and mobility, fracture, and weighted CCI were assessed at any time before the index date; recent surgery was assessed within 1 year before the index date; sarcopenia was assessed using the latest available measurements from instances 0–3 before the index date.

Abbreviations: BMI, body mass index; CCI, Charlson comorbidity index; IQR, interquartile range; SD, standard deviation.

^a^ We categorized participants into three mutually exclusive groups, specifically low (< 600 metabolic equivalent (MET)‐min/week), moderate (600 to < 3000 MET‐min/week) and high (≥ 3000 MET‐min/week) level of physical activity, based on a standard scoring criterion.

**FIGURE 2:**
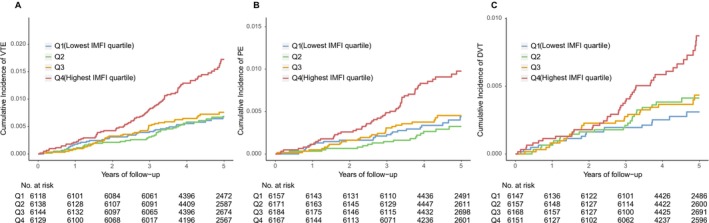
Time to incident (A) VTE, (B) PE and (C) DVT by quartiles of IMFI. DVT, deep vein thrombosis; PE, pulmonary embolism; VTE, venous thromboembolism.

**FIGURE 3:**
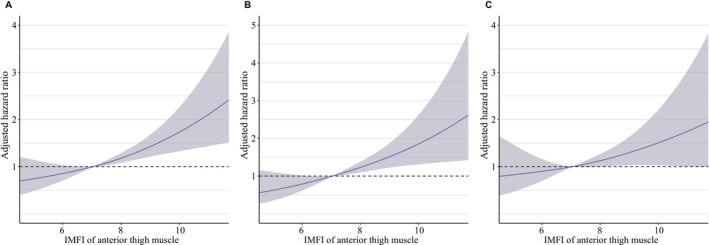
Restricted cubic spline regression models assessing the dose–response associations between IMFI and incident (A) VTE, (B) PE, and (C) DVT. Models were adjusted for age, sex, BMI, educational attainment, smoking status, alcohol consumption, physical activity levels, white ethnicity, frailty status, weighted CCI, recent surgery, abnormalities of gait and mobility, fracture, sarcopenia and use of anticoagulant, glucocorticoid, aspirin and hormone therapy. IMFI, intramuscular fat infiltration. *p* for nonlinearity = 0.733 (VTE), 0.886 (PE) and 0.779 (DVT).

Over a mean follow‐up of 4.92 years (SD: 1.26), 227 participants developed VTE. Participants in the highest IMFI quartile had a markedly higher cumulative incidence of VTE than those in the lower quartiles (Figure 2A). The incidence rates of VTE were 1.34, 1.28, 1.58 and 3.34 per 1000 person‐years in the first (reference), second, third and fourth IMFI quartiles, respectively (Table 2). The corresponding rate differences compared with the reference quartile were −0.05 (95% CI: −0.63, 0.52), 0.24 (95% CI: −0.37, 0.85) and 2.00 (95% CI: 1.23, 2.77) per 1000 person‐years for the second, third and fourth quartiles, respectively. The crude HRs for incident VTE were 0.96 (95% CI: 0.62, 1.49), 1.18 (95% CI: 0.77, 1.79) and 2.49 (95% CI: 1.72, 3.59) for the second, third and fourth quartiles of IMFI, respectively, compared with the reference group. After multivariable adjustment, the HRs for incident VTE were 0.88 (95% CI: 0.57, 1.37), 1.02 (95% CI: 0.67, 1.55) and 1.88 (95% CI: 1.26, 2.80) in the second, third and fourth quartiles of IMFI, respectively. The *E* value for the association between the highest IMFI quartile and incident VTE was 3.17 (95% CI: 1.83, 5.04), indicating that an unmeasured confounder would need to be associated with both IMFI and VTE risk by a risk ratio of at least 3.17 each, above and beyond the measured covariates, to fully explain away the observed association. Restricted cubic spline regression showed a clear increase in VTE risk with higher IMFI levels, with no evidence of nonlinearity (*p* for nonlinearity = 0.733) (Figure 3A).

**TABLE 2:** Association between IMFI in the anterior thigh and incident VTE, PE and DVT.

	IMFI in the anterior thigh at baseline			
	Q1 (lowest)^a^	Q2	Q3	Q4 (highest)
Incident VTE				
Number of participants, *n*	6118	6138	6144	6129
Incident cases, *n*	40	39	48	100
Mean follow‐up time (SD), years	4.89 (1.22)	4.95 (1.26)	4.95 (1.27)	4.89 (1.30)
Incidence rate (per 1000 person‐years)	1.34	1.28	1.58	3.34
Rate difference (95% CI), per 1000 person‐years	0.0 (reference)	−0.05 (−0.63, 0.52)	0.24 (−0.37, 0.85)	2.00 (1.23, 2.77)
Crude HR (95% CI)	1.0 (reference)	0.96 (0.62, 1.49)	1.18 (0.77, 1.79)	2.49 (1.72, 3.59)
Adjusted HR^b^ (95% CI)	1.0 (reference)	0.88 (0.57, 1.37)	1.02 (0.67, 1.55)	1.88 (1.26, 2.80)
Adjusted HR (95% CI) after missing data imputation	1.0 (reference)	0.88 (0.57, 1.37)	1.02 (0.67, 1.55)	1.87 (1.25, 2.79)
Incident PE				
Number of participants, *n*	6157	6171	6184	6167
Incident cases, *n*	26	17	29	62
Mean follow‐up time (SD), years	4.89 (1.21)	4.96 (1.26)	4.96 (1.26)	4.90 (1.29)
Incidence rate (per 1000 person‐years)	0.86	0.56	0.95	2.05
Rate difference (95% CI), per 1000 person‐years	0.0 (reference)	−0.31 (−0.74, 0.12)	0.08 (−0.40, 0.56)	1.19 (0.58, 1.80)
Crude HR (95% CI)	1.0 (reference)	0.64 (0.35, 1.18)	1.09 (0.64, 1.86)	2.37 (1.50, 3.74)
Adjusted HR (95% CI)	1.0 (reference)	0.59 (0.32, 1.08)	0.96 (0.57, 1.62)	1.80 (1.08, 3.01)
Adjusted HR (95% CI) after missing data imputation	1.0 (reference)	0.58 (0.32, 1.07)	0.95 (0.56, 1.61)	1.77 (1.06, 2.97)
Incident DVT				
Number of participants, *n*	6147	6157	6168	6151
Incident cases, *n*	18	26	28	46
Mean follow‐up time (SD), years	4.89 (1.21)	4.96 (1.26)	4.96 (1.26)	4.91 (1.29)
Incidence rate (per 1000 person‐years)	0.60	0.85	0.92	1.52
Rate difference (95% CI), per 1000 person‐years	0.0 (reference)	0.25 (−0.17, 0.68)	0.32 (−0.12, 0.76)	0.92 (0.41, 1.45)
Crude HR (95% CI)	1.0 (reference)	1.43 (0.78, 2.60)	1.53 (0.85, 2.76)	2.54 (1.47, 4.37)
Adjusted HR (95% CI)	1.0 (reference)	1.33 (0.73, 2.43)	1.30 (0.70, 2.39)	1.88 (1.05, 3.37)
Adjusted HR (95% CI) after missing data imputation	1.0 (reference)	1.33 (0.72, 2.43)	1.31 (0.71, 2.41)	1.89 (1.06, 3.40)

Abbreviations: CI, confidence interval; DVT, deep vein thrombosis; HR, hazard ratio; IMFI, intramuscular fat infiltration; PE, pulmonary embolism; SD, standard deviation; VTE, venous thromboembolism.

^a^ Q1–Q4 represent quartiles of IMFI in the anterior thigh at baseline. The quartile ranges for each outcome are as follows: for incident VTE: Q1 (3.17–7.70), Q2 (4.67–8.65), Q3 (5.36–10.12), Q4 (6.21–27.49); for incident PE: Q1 (3.17–7.70), Q2 (4.67–8.65), Q3 (5.36–10.12), Q4 (6.21–27.49); for incident DVT: Q1 (3.17–7.70), Q2 (4.67–8.70), Q3 (5.36–10.12), Q4 (6.21–27.49).

^b^ Adjusted for age, sex, BMI, educational attainment, smoking status, alcohol consumption, physical activity levels, white ethnicity, frailty status, weighted CCI, recent surgery, abnormalities of gait and mobility, fracture, sarcopenia, and use of anticoagulant, glucocorticoid, aspirin and hormone therapy.

During the follow‐up period, 134 participants developed PE. Participants in the highest IMFI quartile had the highest cumulative incidence of PE (Figure 2B). The incidence rates of PE were 0.86, 0.56, 0.95 and 2.05 per 1000 person‐years across increasing IMFI quartiles (Table 2). Compared with the reference quartile, the corresponding rate differences were −0.31 (95% CI: −0.74, 0.12), 0.08 (95% CI: −0.40, 0.56) and 1.19 (95% CI: 0.58, 1.80) per 1000 person‐years for the second through fourth quartiles, respectively. The crude HRs for incident PE in the second, third and fourth quartiles were 0.64 (95% CI: 0.35, 1.18), 1.09 (95% CI: 0.64, 1.86) and 2.37 (95% CI: 1.50, 3.74), respectively, compared with the reference group. After multivariable adjustment, the HRs for PE were 0.59 (95% CI: 0.32, 1.08), 0.96 (95% CI: 0.57, 1.62) and 1.80 (95% CI: 1.08, 3.01) for the second, third and fourth quartiles, respectively. The *E* value for the association between the highest IMFI quartile and incident PE was 3.00 (95% CI: 1.37, 5.47). Restricted cubic spline regression showed a pattern similar to that observed for VTE (*p* for nonlinearity = 0.886) (Figure 3B).

A total of 118 participants developed DVT during the follow‐up period. Similar to VTE and PE, higher IMFI quartiles were associated with higher cumulative incidence of DVT (Figure 2C). As shown in Table 2, the incidence rates of DVT were 0.60, 0.85, 0.92 and 1.52 per 1000 person‐years across increasing IMFI quartiles, with absolute rate differences of 0.25 (95% CI: −0.17, 0.68), 0.32 (95% CI: −0.12, 0.76) and 0.92 (95% CI: 0.41, 1.45) per 1000 person‐years for the second, third and fourth quartiles, respectively, compared with the reference group. The crude HRs for incident DVT were 1.43 (95% CI: 0.78, 2.60), 1.53 (95% CI: 0.85, 2.76) and 2.54 (95% CI: 1.47, 4.37) in the second, third and fourth quartiles, respectively, compared with the reference group. After adjustment for potential confounders, the HRs were 1.33 (95% CI: 0.73, 2.43), 1.30 (95% CI: 0.70, 2.39) and 1.88 (95% CI: 1.05, 3.37) for the second, third and fourth quartiles, respectively. The corresponding *E* value for incident DVT was 3.17 (95% CI: 1.28, 6.20). Restricted cubic spline regression showed a pattern similar to that observed for VTE and PE (*p* for nonlinearity = 0.779) (Figure 3C). The proportional hazards assumption was not violated for VTE, PE or DVT models (all *p* values > 0.05). Results from the sensitivity analyses using multiple imputations were consistent with the primary findings for VTE, PE and DVT (Table 2).

Analyses using IMFI measured in the posterior thigh showed results generally consistent with the primary analyses based on anterior thigh IMFI. Compared with the lowest quartile, the highest quartile of posterior thigh IMFI was associated with significantly increased risks of incident VTE (HR: 1.76; 95% CI: 1.18, 2.62), PE (HR: 1.77; 95% CI: 1.07, 2.94) and DVT (HR: 1.84; 95% CI: 1.03, 3.28) after multivariable adjustment (Table S4). Subgroup analyses stratified by sex, age and BMI showed results consistent with the main analyses (Tables S5–S7). Participants in the highest IMFI quartile generally had higher risks of VTE, PE and DVT across subgroups, although some associations were not statistically significant, likely due to smaller sample sizes.

## 4 Discussion

In this large population‐based cohort study, higher levels of thigh IMFI were significantly associated with an increased risk of VTE, including PE and DVT. These findings suggest that elevated thigh IMFI may represent a modifiable risk factor for VTE, highlighting its potential relevance in VTE risk stratification and prevention strategies.

VTE is a multifactorial condition, influenced by several well‐established risk factors such as cancer, trauma and immobility [3]. Previous studies have mainly focused on general or central adiposity, such as BMI and visceral fat, in relation to VTE risk. Higher BMI and obesity have been associated with an increased risk of VTE across both sex and age groups, and population‐based data suggest that a considerable proportion of VTE events can be attributed to excess body weight, particularly among individuals over 50 years of age [22]. Visceral adiposity and its related metabolic score have also been shown to be independently associated with higher risks of VTE, PE and DVT [23], and greater visceral adipose tissue accumulation has been linked to elevated coagulation factors and enhanced thrombin generation [24]. Additionally, adiposity in other locations, such as hepatic fat accumulation, has been associated with VTE risk [25]. IMFI, a form of ectopic fat infiltration within skeletal muscle, may share similar pathophysiological mechanisms. However, its relationship with VTE risk has not been previously explored. To our knowledge, this study provides large‐scale, population‐based evidence of a direct association between thigh IMFI and incident VTE, reinforcing the potential role of muscle fat infiltration in thrombotic risk.

Several biological mechanisms may explain the observed association between IMFI and VTE risk. First, IMFI is known to induce metabolic and inflammatory dysregulation [6]. Lipid accumulation within myocytes disrupts mitochondrial function, impairs β‐oxidation and increases reactive oxygen species production, collectively contributing to insulin resistance and systemic inflammation [6]. Pro‐inflammatory factors further promote immune cell infiltration and cytokine release [26], amplifying metabolic dysfunction. Second, elevated skeletal IMFI levels are associated with reduced muscle strength and mobility [8], potentially leading to venous stasis, a key element in Virchow's triad for thrombogenesis [27]. Adipose‐derived cytokines and lipid metabolites may exacerbate muscle force weakness and disrupt neuromuscular activation, compromising contractile function [28]. Given that venous return is largely facilitated by the skeletal muscle pump [29], impaired pump function may predispose individuals to venous insufficiency and stasis [30]. Recent experimental evidence further supports this concept by demonstrating that increased intramuscular adipose tissue accumulation can impair ischemic limb muscle strength, whereas preventing its formation improves muscle function [31].

Our findings have several potential clinical and research implications. Although anatomically localized, thigh imaging has been widely used in muscle research to characterize adverse muscle composition, and thigh IMFI may reflect broader systemic processes related to skeletal muscle quality, metabolic dysfunction and overall health status [14, 32]. Importantly, as a form of ectopic fat infiltration within skeletal muscle, IMFI may capture tissue‐specific alterations not fully reflected by conventional adiposity measures and contribute to venous thromboembolism through mechanisms beyond systemic effects. Increased intramuscular fat infiltration likely contributes to local effects by impairing skeletal muscle function and reducing muscle pump efficiency, as well as by promoting a prothrombotic microenvironment that increases thrombosis susceptibility. Our findings suggest that IMFI may represent a potentially modifiable component associated with thrombotic risk, highlighting its relevance for preventive strategies targeting adverse muscle fat accumulation. Lifestyle‐based interventions, such as resistance training and nutritional supplementation (e.g., omega‐3 fatty acids and HMB) [10, 11, 33], as well as pharmacologic approaches, including glucagon‐like peptide‐1 receptor agonists [34], may help reduce IMFI. Targeted modulation of the adipose‐related intramuscular microenvironment has recently been proposed as a potential strategy to improve outcomes associated with adverse muscle fat accumulation [35]. Given the rising prevalence of sarcopenic obesity and age‐related muscle fat infiltration, interventions aimed at reducing IMFI may hold promise for mitigating thrombotic risk. Furthermore, while MRI is considered the gold standard for assessing muscle fat infiltration, IMFI can also be reliably estimated using ultrasound [36], a widely available and routinely used modality in VTE diagnosis [3]. Notably, studies have demonstrated strong correlations between ultrasound‐ and MRI‐derived IMFI measurements, supporting its feasibility for clinical assessment [37]. Assessment of IMFI may be particularly relevant in individuals with normal BMI but impaired muscle quality, whose thrombotic risk may not be fully captured by conventional adiposity measures. Together, these findings support further investigation of IMFI as a clinically accessible marker of muscle quality and thrombotic risk.

Our study has several strengths. In this study, thigh IMFI was accurately quantified using MRI, ensuring consistency across different MR scanner manufacturers (GE, Philips and Siemens) and magnetic field strengths (1.5 and 3 T) [38]. In addition, we adjusted for key confounders to minimize bias and enhance the validity of our findings. Specifically, we used age‐ and sex‐specific IMFI quartiles to improve comparability across individuals and better control the strong confounding effects of these two factors.

### 4.1 Limitations

This study has several limitations. First, the UK Biobank cohort predominantly consists of individuals of European ancestry, which may limit the generalizability of our findings to other ethnic groups. Second, although we adjusted for multiple confounders, residual confounding cannot be entirely ruled out, and causality cannot be inferred from this observational study. While a temporal association between IMFI and incident VTE was established, the findings should be interpreted with caution, as they may not fully reflect causal relationships in clinical settings. Third, IMFI was assessed only in the thigh region, and fat infiltration patterns may vary across different muscle groups. Therefore, the findings may not fully capture IMFI distribution in other skeletal muscle regions or its broader clinical relevance.

### 4.2 Future Directions

Future studies are needed to further clarify the temporal and potential causal relationship between IMFI and VTE. In particular, interventional studies may help determine whether reductions in IMFI are associated with subsequent decreases in VTE risk. Experimental studies are also warranted to elucidate the biological mechanisms linking IMFI to thrombogenesis. From a translational perspective, genetic and molecular investigations could uncover novel pathways through which ectopic fat deposition in skeletal muscle contributes to prothrombotic states, potentially identifying new targets for therapeutic intervention.

## 5 Conclusions

In this large population‐based cohort study, elevated thigh IMFI was associated with an increased risk of VTE. These findings suggest that high thigh IMFI may represent a potentially modifiable risk factor for VTE. Further research is warranted to elucidate the underlying mechanisms and clarify the causal relationship, as well as to guide prevention strategies and inform clinical decision‐making in thrombotic disease.

## Funding

This work was supported by the National Key Research and Development Project (GL: 2022YFC3601900, CZ: 2022YFC2505500), the National Natural Science Foundation of China (GL: U21A20352, CZ: 82072502, CZ: 82372474, YW: 82302771), the Project Program of National Clinical Research Center for Geriatric Disorders (JW: 2021LNJJ06, CZ: 2022LNJJ07, YW: 2023LNJJ17), the Central South University Innovation‐Driven Research Programme (CZ: 2023CXQD031), the Science and Technology Innovation Program of Hunan Province (JW: 2022RC1009, CZ: 2022RC3075, YW: 2024RC3049), the Scientific Research Program of FuRong Laboratory (JW: 2023SK2100, GL: 2024PT5108), the Excellent Youth Science Foundation of Xiangya Hospital, Central South University (YW: 2024YQ01), and the Scientific Research Program of the Education Department of Hunan Province (YW: 296840).

## Ethics Statement

The UK Biobank was approved by the National Health Service and the National Research Ethics Service (reference 11/NW/0382). All participants provided written informed consent before taking part.

## Conflicts of Interest

The authors declare no conflicts of interest.

## Transparency

The lead author affirms that the manuscript is an honest, accurate and transparent account of the study being reported; that no important aspects of the study have been omitted; and that any discrepancies from the study as planned have been explained.

## Dissemination to Participants and Related Patient and Public Communities

Findings from this study will be disseminated through the websites of the authors' institutes alongside the publication of this manuscript. Social media will be used to draw attention to the work and stimulate debate about its findings.

## Supporting information


**Table S1:** ICD codes for clinical outcomes.
**Table S2: Baseline characteristics of participants in PE analysis,** ^a^ We categorized participants into three mutually exclusive groups, specifically low (< 600 metabolic equivalent (MET)‐min/week), moderate (600 to < 3000 MET‐min/week), and high (≥ 3000 MET‐min/week) level of physical activity, based on a standard scoring criterion.
**Table S3:** Baseline characteristics of participants in DVT analysis.
**Table S4:** Association between IMFI in the posterior thigh and incident VTE, PE, and DVT.
**Table S5:** Association between IMFI in the anterior thigh and incident VTE, PE, and DVT by sex.
**Table S6:** Association between IMFI in the anterior thigh and incident VTE, PE, and DVT by age.
**Table S7:** Association between IMFI in the anterior thigh and incident VTE, PE, and DVT by BMI.

## Data Availability

The data and analytical code used in this study are available from the corresponding author upon reasonable request.
