# Supplementary material for: Association of Intramuscular Fat Infiltration With Incident Venous Thromboembolism: A Population‐Based Cohort Study
Source: J Cachexia Sarcopenia Muscle. 2026 Jul 9;17(4):e70342. doi: 10.1002/jcsm.70342 (PMC13347145; doi:10.1002/jcsm.70342)
Supplement: Supplementary file 1 — Table S1: ICD codes for clinical outcomes. Table S2: Baseline characteristics of participants in PE analysis, a We categorized participants into three mutually exclusive groups, specifically low (< 600 metabolic equivalent (MET)‐min/week), moderate (600 to < 3000 MET‐min/week), and high (≥ 3000 MET‐min/week) level of physical activity, based on a standard scoring criterion. Table S3: Baseline characteristics of participants in DVT analysis. Table S4: Association between IMFI in the posterior thigh and incident VTE, PE, and DVT. Table S5: Association between IMFI in the anterior thigh and incident VTE, PE, and DVT by sex. Table S6: Association between IMFI in the anterior thigh and incident VTE, PE, and DVT by age. Table S7: Association between IMFI in the anterior thigh and incident VTE, PE, and DVT by BMI. [file JCSM-17-e70342-s001.docx]

**Table S1. ICD codes for clinical outcomes**

| **Clinical outcomes** | **ICD-9 diagnosis** | **ICD-10 diagnosis** |
| --- | --- | --- |
| **Venous thromboembolism** | 4151, 4511 | I26, I260, I269, I800, I801, I802, I803, I808, I809, I81, I82, I820, I822, D68, D680, D681, D682, D683, D684, D685, D686, D688, D689 |
| **Pulmonary embolism** | 4151 | I26, I260, I269 |
| **Deep vein thrombosis** | 4511 | I800, I801, I802, I803, I808, I809, I81, I82, I820, I822, D68, D680, D681, D682, D683, D684, D685, D686, D688, D689 |

**Table S2. Baseline characteristics of participants in PE analysis**

|  | **Age- and sex-specific quartile of intramuscular fat infiltration of anterior thigh** | | | | **Standardized mean difference** |
| --- | --- | --- | --- | --- | --- |
|  | **Q1 (lowest)**  **(3.17-7.70)** | **Q2**  **(4.67-8.65)** | **Q3**  **(5.36-10.12)** | **Q4 (highest)**  **(6.21-27.49)** |  |
| **Participants, n** | 6,157 | 6,171 | 6,184 | 6,167 |  |
| **Age, years (median [IQR])** | 56 (49-61) | 56 (49-61) | 56 (49-61) | 56 (49-61) | 0.001 |
| **Sex (male), n (%)** | 2,950 (47.9) | 2,955 (47.9) | 2,964 (47.9) | 2,954 (47.9) | <0.001 |
| **BMI, kg/m^2^ (median [IQR])** | 24.03 (22.26-26.08) | 25.44 (23.37-27.65) | 26.62 (24.41-29.11) | 28.85 (26.23-32.10) | 0.725 |
| **Education, n (%)** |  |  |  |  | 0.204 |
| Higher education | 4,203 (68.3) | 3,887 (63.0) | 3,534 (57.1) | 3,190 (51.7) |  |
| Upper secondary | 409 (6.6) | 512 (8.3) | 524 (8.5) | 518 (8.4) |  |
| Lower secondary | 951 (15.4) | 1,033 (16.7) | 1,228 (19.9) | 1,411 (22.9) |  |
| Vocational | 225 (3.7) | 294 (4.8) | 328 (5.3) | 332 (5.4) |  |
| Other | 278 (4.5) | 351 (5.7) | 434 (7.0) | 576 (9.3) |  |
| Missing | 91 (1.5) | 94 (1.5) | 136 (2.2) | 140 (2.3) |  |
| **Smoking, n (%)** |  |  |  |  | 0.172 |
| Current | 250 (4.1) | 363 (5.9) | 422 (6.8) | 535 (8.7) |  |
| Previous | 1,754 (28.5) | 1,896 (30.7) | 2,124 (34.4) | 2,332 (37.8) |  |
| Never | 4,143 (67.3) | 3,901 (63.2) | 3,624 (58.6) | 3,278 (53.2) |  |
| Missing | 10 (0.2) | 11 (0.2) | 14 (0.2) | 22 (0.4) |  |
| **Alcohol, n (%)** |  |  |  |  | 0.029 |
| Current | 5,868 (95.3) | 5,865 (95.1) | 5,873 (95.0) | 5,849 (94.9) |  |
| Previous | 132 (2.1) | 138 (2.2) | 127 (2.1) | 151 (2.4) |  |
| Never | 156 (2.5) | 166 (2.7) | 178 (2.9) | 163 (2.6) |  |
| Missing | 1 (0.0) | 2 (0.0) | 6 (0.1) | 4 (0.1) |  |
| **Physical activity level^a^, n (%)** |  |  |  |  | 0.180 |
| Low | 692 (11.2) | 881 (14.3) | 1,037 (16.8) | 1,295 (21.0) |  |
| Moderate | 2,928 (47.6) | 2,886 (46.8) | 2,779 (44.9) | 2,551 (41.4) |  |
| High | 1,691 (27.5) | 1,534 (24.9) | 1,330 (21.5) | 1,252 (20.3) |  |
| Missing | 846 (13.7) | 870 (14.1) | 1,038 (16.8) | 1,069 (17.3) |  |
| **White ethnic, n (%)** | 5,992 (97.3) | 5,989 (97.1) | 5,977 (96.7) | 5,989 (97.1) | 0.020 |
| **Frailty status, n (%)** |  |  |  |  | 0.244 |
| Robust | 4,262 (69.2) | 3,944 (63.9) | 3,492 (56.5) | 3,038 (49.3) |  |
| Pre-frail | 1,394 (22.6) | 1,637 (26.5) | 1,995 (32.3) | 2,265 (36.7) |  |
| Frail | 15 (0.2) | 44 (0.7) | 49 (0.8) | 124 (2.0) |  |
| Missing | 486 (7.9) | 546 (8.8) | 648 (10.5) | 740 (12.0) |  |
| **Weighted CCI, n (%)** |  |  |  |  | 0.135 |
| CCI=0 | 5,330 (86.6) | 5,144 (83.4) | 5,003 (80.9) | 4,753 (77.1) |  |
| CCI>0 | 827 (13.4) | 1,027 (16.6) | 1,181 (19.1) | 1,414 (22.9) |  |
| **Recent surgery, n (%)** | 628 (10.2) | 716 (11.6) | 767 (12.4) | 884 (14.3) | 0.067 |
| **Abnormalities of gait and mobility, n (%)** | 3 (0.0) | 6 (0.1) | 12 (0.2) | 15 (0.2) | 0.030 |
| **Fracture, n (%)** | 105 (1.7) | 108 (1.8) | 122 (2.0) | 137 (2.2) | 0.021 |
| **Sarcopenia, n (%)** | 44 (0.7) | 26 (0.4) | 24 (0.4) | 15 (0.2) | 0.036 |
| **Medication, n (%)** |  |  |  |  |  |
| Anticoagulant | 26 (0.4) | 32 (0.5) | 48 (0.8) | 84 (1.4) | 0.056 |
| Glucocorticoid | 150 (2.4) | 187 (3.0) | 198 (3.2) | 264 (4.3) | 0.053 |
| Aspirin | 536 (8.7) | 583 (9.4) | 692 (11.2) | 815 (13.2) | 0.082 |
| Hormone therapy | 1,014 (16.5) | 1,072 (17.4) | 1,122 (18.1) | 1,155 (18.7) | 0.033 |
| **Death, n (%)** | 70 (1.1) | 85 (1.4) | 104 (1.7) | 131 (2.1) | 0.043 |

IQR, interquartile range; BMI, body mass index; SD, standard deviation; IMFI, intramuscular fat infiltration; CCI, Charlson comorbidity index (including cancer, cerebrovascular disease, chronic obstructive pulmonary disease, dementia, diabetes, heart failure, myocardial infarction, hemiplegia, AIDS, liver disease, chronic kidney disease, other chronic pulmonary disease, peripheral vascular disease, rheumatic diseases, and peptic ulcer disease) was calculated with scores ranging from 0 to 10. For analysis, Weighted CCI was dichotomized into two groups: 0 (no comorbidity burden) and >0 (any comorbidity burden).

Age, sex, BMI, education, smoking, alcohol, physical activity level, white ethnicity, frailty status, and medication use were assessed at the initial assessment visit (instance 0); abnormalities of gait and mobility, fracture, and weighted CCI were assessed at any time before the index date; recent surgery was assessed within 1 year before the index date; sarcopenia was assessed using the latest available measurements from instances 0-3 before the index date.

^a^ We categorised participants into three mutually exclusive groups, specifically low (<600 metabolic equivalent (MET)-min/week), moderate (600 to <3000 MET-min/week), and high (≥3000 MET-min/week) level of physical activity, based on a standard scoring criterion.**Table S3. Baseline characteristics of participants in DVT analysis**

|  | **Age- and sex-specific quartile of intramuscular fat infiltration of anterior thigh** | | | | **Standardized mean difference** |
| --- | --- | --- | --- | --- | --- |
|  | **Q1 (lowest)**  **(3.17-7.70)** | **Q2**  **(4.67-8.70)** | **Q3**  **(5.36-10.12**) | **Q4 (highest)**  **(6.20-27.49)** |  |
| **Participants, n** | 6,147 | 6,157 | 6,168 | 6,151 |  |
| **Age, years (median [IQR])** | 56 (49-61) | 56 (49-61) | 56 (49-61) | 56 (49-61) | 0.002 |
| **Sex (male), n (%)** | 2,946 (47.9) | 2,950 (47.9) | 2,957 (47.9) | 2,949 (47.9) | <0.001 |
| **BMI, kg/m^2^ (median [IQR])** | 24.02 (22.25-26.08) | 25.43 (23.37-27.65) | 26.62 (24.41-29.12) | 28.85 (26.22-32.10) | 0.725 |
| **Education, n (%)** |  |  |  |  | 0.204 |
| Higher education | 4,194 (68.2) | 3,878 (63.0) | 3,527 (57.2) | 3,182 (51.7) |  |
| Upper secondary | 407 (6.6) | 514 (8.3) | 518 (8.4) | 511 (8.3) |  |
| Lower secondary | 953 (15.5) | 1,030 (16.7) | 1,230 (19.9) | 1,407 (22.9) |  |
| Vocational | 225 (3.7) | 294 (4.8) | 325 (5.3) | 333 (5.4) |  |
| Other | 276 (4.5) | 347 (5.6) | 433 (7.0) | 577 (9.4) |  |
| Missing | 92 (1.5) | 94 (1.5) | 135 (2.2) | 141 (2.3) |  |
| **Smoking, n (%)** |  |  |  |  | 0.174 |
| Current | 250 (4.1) | 362 (5.9) | 423 (6.9) | 535 (8.7) |  |
| Previous | 1,752 (28.5) | 1,883 (30.6) | 2,130 (34.5) | 2,330 (37.9) |  |
| Never | 4,135 (67.3) | 3,901 (63.4) | 3,601 (58.4) | 3,264 (53.1) |  |
| Missing | 10 (0.2) | 11 (0.2) | 14 (0.2) | 22 (0.4) |  |
| **Alcohol, n (%)** |  |  |  |  | 0.029 |
| Current | 5,863 (95.4) | 5,853 (95.1) | 5,857 (95.0) | 5,834 (94.9) |  |
| Previous | 130 (2.1) | 135 (2.2) | 130 (2.1) | 152 (2.5) |  |
| Never | 153 (2.5) | 167 (2.7) | 175 (2.8) | 161 (2.6) |  |
| Missing | 1 (0.0) | 2 (0.0) | 6 (0.1) | 4 (0.1) |  |
| **Physical activity level^a^, n (%)** |  |  |  |  | 0.182 |
| Low | 689 (11.2) | 878 (14.3) | 1,036 (16.8) | 1,295 (21.1) |  |
| Moderate | 2,926 (47.6) | 2,882 (46.8) | 2,773 (45.0) | 2,535 (41.2) |  |
| High | 1,689 (27.5) | 1,528 (24.8) | 1,326 (21.5) | 1,251 (20.3) |  |
| Missing | 843 (13.7) | 869 (14.1) | 1,033 (16.7) | 1,070 (17.4) |  |
| **White ethnic, n (%)** | 5,983 (97.3) | 5,974 (97.0) | 5,964 (96.7) | 5,974 (97.1) | 0.020 |
| **Frailty status, n (%)** |  |  |  |  | 0.243 |
| Robust | 4,254 (69.2) | 3,938 (64.0) | 3,487 (56.5) | 3,035 (49.3) |  |
| Pre-frail | 1,393 (22.7) | 1,632 (26.5) | 1,986 (32.2) | 2,251 (36.6) |  |
| Frail | 15 (0.2) | 43 (0.7) | 49 (0.8) | 126 (2.0) |  |
| Missing | 485 (7.9) | 544 (8.8) | 646 (10.5) | 739 (12.0) |  |
| **Weighted CCI, n (%)** |  |  |  |  | 0.133 |
| CCI=0 | 5,318 (86.5) | 5,135 (83.4) | 4,994 (81.0) | 4,747 (77.2) |  |
| CCI>0 | 829 (13.5) | 1,022 (16.6) | 1,174 (19.0) | 1,404 (22.8) |  |
| **Recent surgery, n (%)** | 626 (10.2) | 716 (11.6) | 756 (12.3) | 881 (14.3) | 0.067 |
| **Abnormalities of gait and mobility, n (%)** | 3 (0.0) | 5 (0.1) | 13 (0.2) | 16 (0.3) | 0.033 |
| **Fracture, n (%)** | 107 (1.7) | 107 (1.7) | 119 (1.9) | 136 (2.2) | 0.019 |
| **Sarcopenia, n (%)** | 43 (0.7) | 27 (0.4) | 24 (0.4) | 15 (0.2) | 0.035 |
| **Medication, n (%)** |  |  |  |  |  |
| Anticoagulant | 26 (0.4) | 26 (0.4) | 42 (0.7) | 79 (1.3) | 0.053 |
| Glucocorticoid | 150 (2.4) | 186 (3.0) | 197 (3.2) | 262 (4.3) | 0.052 |
| Aspirin | 536 (8.7) | 582 (9.5) | 697 (11.3) | 813 (13.2) | 0.082 |
| Hormone therapy | 1,012 (16.5) | 1,068 (17.3) | 1,117 (18.1) | 1,151 (18.7) | 0.033 |
| **Death, n (%)** | 71 (1.2) | 82 (1.3) | 108 (1.8) | 131 (2.1) | 0.044 |

IQR, interquartile range; BMI, body mass index; SD, standard deviation; IMFI, intramuscular fat infiltration; CCI, Charlson comorbidity index (including cancer, cerebrovascular disease, chronic obstructive pulmonary disease, dementia, diabetes, heart failure, myocardial infarction, hemiplegia, AIDS, liver disease, chronic kidney disease, other chronic pulmonary disease, peripheral vascular disease, rheumatic diseases, and peptic ulcer disease) was calculated with scores ranging from 0 to 10. For analysis, Weighted CCI was dichotomized into two groups: 0 (no comorbidity burden) and >0 (any comorbidity burden).

Age, sex, BMI, education, smoking, alcohol, physical activity level, white ethnicity, frailty status, and medication use were assessed at the initial assessment visit (instance 0); abnormalities of gait and mobility, fracture, and weighted CCI were assessed at any time before the index date; recent surgery was assessed within 1 year before the index date; sarcopenia was assessed using the latest available measurements from instances 0-3 before the index date.

^a^ We categorised participants into three mutually exclusive groups, specifically low (<600 metabolic equivalent (MET)-min/week), moderate (600 to <3000 MET-min/week), and high (≥3000 MET-min/week) level of physical activity, based on a standard scoring criterion.

**Table S4.** **Association between IMFI in the posterior thigh and incident** **VTE, PE, and DVT**

|  | **IMFI in the posterior thigh at baseline** | | | |
| --- | --- | --- | --- | --- |
|  | **Q1 (lowest)**^a^ | **Q2** | **Q3** | **Q4 (highest)** |
| **Incident VTE** |  |  |  |  |
| Number of participants, n | 6,130 | 6,144 | 6,152 | 6,140 |
| Incident cases, n | 38 | 59 | 45 | 85 |
| Mean follow-up time (SD), years | 4.99 (1.26) | 4.93 (1.26) | 4.92 (1.26) | 4.85 (1.28) |
| Incidence rate (per 1000 person-years) | 1.24 | 1.95 | 1.49 | 2.86 |
| Rate difference (95%CI), per 1000 person-years | 0.0 (reference) | 0.70 (0.07, 1.34) | 0.25 (-0.34, 0.83) | 1.61 (0.89, 2.34) |
| Crude HR (95%CI) | 1.0 (reference) | 1.57 (1.04, 2.35) | 1.19 (0.78, 1.84) | 2.29 (1.56, 3.35) |
| Adjusted HR^b^ (95%CI) | 1.0 (reference) | 1.42 (0.94, 2.15) | 1.01 (0.65, 1.56) | 1.76 (1.18, 2.62) |
| **Incident PE** |  |  |  |  |
| Number of participants, n | 6,166 | 6,184 | 6,194 | 6,172 |
| Incident cases, n | 24 | 31 | 28 | 51 |
| Mean follow-up time (SD), years | 4.99 (1.25) | 4.94 (1.25) | 4.92 (1.26) | 4.86 (1.27) |
| Incidence rate (per 1000 person-years) | 0.78 | 1.01 | 0.92 | 1.70 |
| Rate difference (95%CI), per 1000 person-years | 0.0 (reference) | 0.24 (-0.24, 0.71) | 0.14 (-0.32, 0.60) | 0.93 (0.36, 1.49) |
| Crude HR (95%CI) | 1.0 (reference) | 1.30 (0.77, 2.22) | 1.18 (0.68, 2.03) | 2.18 (1.34, 3.54) |
| Adjusted HR (95%CI) | 1.0 (reference) | 1.20 (0.70, 2.06) | 1.02 (0.59, 1.77) | 1.77 (1.07, 2.94) |
| **Incident DVT** |  |  |  |  |
| Number of participants, n | 6,155 | 6,168 | 6,178 | 6,159 |
| Incident cases, n | 17 | 35 | 25 | 41 |
| Mean follow-up time (SD), years | 5.00 (1.25) | 4.94 (1.26) | 4.92 (1.25) | 4.86 (1.27) |
| Incidence rate (per 1000 person-years) | 0.55 | 1.15 | 0.82 | 1.37 |
| Rate difference (95%CI), per 1000 person-years | 0.0 (reference) | 0.59 (0.13, 1.06) | 0.27 (-0.15, 0.68) | 0.82 (0.32, 1.31) |
| Crude HR (95%CI) | 1.0 (reference) | 2.07 (1.16, 3.70) | 1.48 (0.80, 2.74) | 2.46 (1.40, 4.33) |
| Adjusted HR (95%CI) | 1.0 (reference) | 1.89 (1.05, 3.37) | 1.23 (0.66, 2.28) | 1.84 (1.03, 3.28) |

HR, hazard ratio; CI, confidence interval; SD, standard deviation; VTE, venous thromboembolism; PE, pulmonary embolism; DVT, deep vein thrombosis; IMFI, intramuscular fat infiltration.

^a^ Q1-Q4 represent quartiles of IMFI in the posterior thigh at baseline. The quartile ranges for each outcome are as follows: For incident VTE: Q1 (4.98-11.45), Q2 (7.52-12.74), Q3 (8.57-14.18), Q4 (9.93-25.69); For incident PE: Q1 (4.98-11.45), Q2 (7.52-12.79), Q3 (8.57-14.19), Q4 (9.93-25.69); For incident DVT: Q1 (4.98-11.46), Q2 (7.52-12.79), Q3 (8.55-14.18), Q4 (9.92-25.69).

^b^ Adjusted for age, sex, BMI, educational attainment, smoking status, alcohol consumption, physical activity levels, white ethnicity, frailty status, weighted CCI, recent surgery, abnormalities of gait and mobility, fracture, sarcopenia, and use of anticoagulant, glucocorticoid, aspirin, and hormone therapy.

**Table S5. Association between IMFI in the anterior thigh and incident** **VTE, PE, and DVT by sex**

|  | **IMFI in the anterior thigh at baseline** | | | |
| --- | --- | --- | --- | --- |
|  | **Q1 (lowest)**^a^ | **Q2** | **Q3** | **Q4 (highest)** |
| **Male** |  |  |  |  |
| **Incident VTE** |  |  |  |  |
| Number of participants, n | 2,928 | 2,938 | 2,940 | 2,934 |
| Incident cases, n | 28 | 17 | 30 | 61 |
| Mean follow-up time (SD), years | 4.88 (1.23) | 4.95 (1.26) | 4.92 (1.29) | 4.83 (1.32) |
| Incidence rate (per 1000 person-years) | 1.96 | 1.17 | 2.07 | 4.30 |
| Rate difference (95%CI), per 1000 person-years | 0.0 (reference) | -0.78 (-1.70, 0.13) | 0.11 (-0.92, 1.16) | 2.34 (1.05, 3.65) |
| Crude HR (95%CI) | 1.0 (reference) | 0.60 (0.33, 1.09) | 1.06 (0.63, 1.77) | 2.19 (1.40, 3.42) |
| Adjusted HR^b^ (95%CI) | 1.0 (reference) | 0.57 (0.31, 1.04) | 0.95 (0.56, 1.59) | 1.77 (1.07, 2.92) |
| **Incident PE** |  |  |  |  |
| Number of participants, n | 2,950 | 2,955 | 2,964 | 2,954 |
| Incident cases, n | 19 | 6 | 17 | 38 |
| Mean follow-up time (SD), years | 4.89 (1.22) | 4.96 (1.26) | 4.93 (1.28) | 4.85 (1.31) |
| Incidence rate (per 1000 person-years) | 1.32 | 0.41 | 1.16 | 2.65 |
| Rate difference (95%CI), per 1000 person-years | 0.0 (reference) | -0.91 (-1.59, -0.23) | -0.15 (-0.96, 0.66) | 1.33 (0.31, 2.37) |
| Crude HR (95%CI) | 1.0 (reference) | 0.31 (0.12, 0.78) | 0.88 (0.46, 1.69) | 2.00 (1.16, 3.48) |
| Adjusted HR^b^ (95%CI) | 1.0 (reference) | 0.29 (0.11, 0.75) | 0.82 (0.43, 1.57) | 1.88 (1.07, 3.32) |
| **Incident DVT** |  |  |  |  |
| Number of participants, n | 2,946 | 2,950 | 2,957 | 2,949 |
| Incident cases, n | 12 | 13 | 20 | 28 |
| Mean follow-up time (SD), years | 4.89 (1.21) | 4.95 (1.26) | 4.92 (1.28) | 4.85 (1.31) |
| Incidence rate (per 1000 person-years) | 0.83 | 0.89 | 1.37 | 1.96 |
| Rate difference (95%CI), per 1000 person-years | 0.0 (reference) | 0.06 (-0.61, 0.74) | 0.55 (-0.22, 1.31) | 1.13 (0.27, 1.99) |
| Crude HR (95%CI) | 1.0 (reference) | 1.08 (0.49, 2.36) | 1.65 (0.81, 3.37) | 2.34 (1.19, 4.60) |
| Adjusted HR^b^ (95%CI) | 1.0 (reference) | 1.05 (0.48, 2.29) | 1.47 (0.71, 3.06) | 1.80 (0.87, 3.73) |
| **Female** |  |  |  |  |
| **Incident VTE** |  |  |  |  |
| Number of participants, n | 3,190 | 3,200 | 3,204 | 3,195 |
| Incident cases, n | 12 | 22 | 18 | 39 |
| Mean follow-up time (SD), years | 4.89 (1.22) | 4.96 (1.26) | 4.98 (1.25) | 4.94 (1.28) |
| Incidence rate (per 1000 person-years) | 0.77 | 1.39 | 1.13 | 3.47 |
| Rate difference (95%CI), per 1000 person-years | 0.0 (reference) | 0.62 (-0.11, 1.34) | 0.36 (-0.32, 1.04) | 1.70 (0.81, 2.59) |
| Crude HR (95%CI) | 1.0 (reference) | 1.80 (0.89, 3.64) | 1.47 (0.71, 3.05) | 3.20 (1.68, 6.12) |
| Adjusted HR^b^ (95%CI) | 1.0 (reference) | 1.56 (0.77, 3.15) | 1.16 (0.56, 2.41) | 2.13 (1.06, 4.25) |
| **Incident PE** |  |  |  |  |
| Number of participants, n | 3,207 | 3,216 | 3,220 | 3,213 |
| Incident cases, n | 7 | 11 | 12 | 24 |
| Mean follow-up time (SD), years | 4.90 (1.21) | 4.97 (1.26) | 4.99 (1.24) | 4.95 (1.28) |
| Incidence rate (per 1000 person-years) | 0.45 | 0.69 | 0.75 | 1.51 |
| Rate difference (95%CI), per 1000 person-years | 0.0 (reference) | 0.24 (-0.29, 0.77) | 0.30 (-0.24, 0.84) | 1.06 (0.37, 1.75) |
| Crude HR (95%CI) | 1.0 (reference) | 1.54 (0.60, 3.98) | 1.68 (0.66, 4.26) | 3.38 (1.45, 7.83) |
| Adjusted HR^b^ (95%CI) | 1.0 (reference) | 1.32 (0.52, 3.34) | 1.42 (0.56, 3.58) | 2.51 (1.03, 6.12) |
| **Incident DVT** |  |  |  |  |
| Number of participants, n | 3,201 | 3,207 | 3,211 | 3,202 |
| Incident cases, n | 6 | 13 | 8 | 18 |
| Mean follow-up time (SD), years | 4.90 (1.21) | 4.96 (1.26) | 4.99 (1.24) | 4.96 (1.28) |
| Incidence rate (per 1000 person-years) | 0.38 | 0.82 | 0.50 | 1.13 |
| Rate difference (95%CI), per 1000 person-years | 0.0 (reference) | 0.44 (-0.17, 0.68) | 0.12 (-0.12, 0.76) | 0.75 (0.41, 1.45) |
| Crude HR (95%CI) | 1.0 (reference) | 2.13 (0.81, 5.60) | 1.30 (0.45, 3.74) | 2.95 (1.17, 7.42) |
| Adjusted HR^b^ (95%CI) | 1.0 (reference) | 1.95 (0.74, 5.12) | 1.07 (0.36, 3.13) | 2.25 (0.92, 5.50) |

HR, hazard ratio; CI, confidence interval; SD, standard deviation; VTE, venous thromboembolism; PE, pulmonary embolism; DVT, deep vein thrombosis; IMFI, intramuscular fat infiltration.

^a^ Q1-Q4 represent quartiles of IMFI in the anterior thigh at baseline. The quartile ranges for each outcome are as follows: For incident VTE: for male: Q1 (3.17-6.56), Q2 (4.67-7.73), Q3 (5.36-9.00), Q4 (6.21-26.40); for female: Q1 (3.50-7.70), Q2 (5.61-8.65), Q3 (6.42-10.12), Q4 (7.33-27.49); For incident PE: for male: Q1 (3.17-6.57), Q2 (4.67-7.74), Q3 (5.36-8.98), Q4 (6.21-26.40); for female: Q1 (3.50-7.70), Q2 (5.61-8.65), Q3 (6.42-10.12), Q4 (7.36-27.49); For incident DVT: for male: Q1 (3.17-6.57), Q2 (4.67-7.74), Q3 (5.36-9.00), Q4 (6.20-26.40); for female: Q1 (3.50-7.70), Q2 (5.61-8.70), Q3 (6.42-10.12), Q4 (7.35-27.49).

^b^ Adjusted for age, sex, BMI, smoking status, alcohol consumption, frailty status, white ethnicity, educational attainment, physical activity levels, weighted CCI, recent surgery, abnormalities of gait and mobility, fracture, sarcopenia, and use of anticoagulant, glucocorticoid, aspirin, and hormone therapy.

**Table S6. Association between IMFI in the anterior thigh and incident** **VTE, PE, and DVT by age**

|  | **IMFI in the anterior thigh at baseline** | | | |
| --- | --- | --- | --- | --- |
|  | **Q1 (lowest)**^a^ | **Q2** | **Q3** | **Q4 (highest)** |
| **Age<60 years** |  |  |  |  |
| **Incident VTE** |  |  |  |  |
| Number of participants, n | 4,149 | 4,161 | 4,165 | 4,155 |
| Incident cases, n | 18 | 23 | 20 | 55 |
| Mean follow-up time (SD), years | 4.86 (1.21) | 4.92 (1.24) | 4.95 (1.23) | 4.88 (1.28) |
| Incidence rate (per 1000 person-years) | 0.89 | 1.12 | 0.97 | 2.71 |
| Rate difference (95%CI), per 1000 person-years | 0.0 (reference) | 0.23 (-0.39, 0.85) | 0.07 (-0.52, 0.67) | 1.82 (0.99, 2.65) |
| Crude HR (95%CI) | 1.0 (reference) | 1.26 (0.68, 2.33) | 1.08 (0.57, 2.05) | 3.03 (1.78, 5.16) |
| Adjusted HR^b^ (95%CI) | 1.0 (reference) | 1.19 (0.64, 2.20) | 0.98 (0.51, 1.87) | 2.47 (1.41, 4.32) |
| **Incident PE** |  |  |  |  |
| Number of participants, n | 4,171 | 4,182 | 4,188 | 4,179 |
| Incident cases, n | 14 | 9 | 13 | 39 |
| Mean follow-up time (SD), years | 4.86 (1.20) | 4.93 (1.23) | 4.96 (1.23) | 4.89 (1.27) |
| Incidence rate (per 1000 person-years) | 0.69 | 0.44 | 0.63 | 1.91 |
| Rate difference (95%CI), per 1000 person-years | 0.0 (reference) | -0.26 (-0.72, 0.21) | -0.07 (-0.56, 0.43) | 1.22 (0.52, 1.92) |
| Crude HR (95%CI) | 1.0 (reference) | 0.63 (0.27, 1.46) | 0.91 (0.43, 1.93) | 2.76 (1.50, 5.08) |
| Adjusted HR^b^ (95%CI) | 1.0 (reference) | 0.58 (0.26, 1.33) | 0.78 (0.37, 1.67) | 2.03 (1.04, 3.96) |
| **Incident DVT** |  |  |  |  |
| Number of participants, n | 4,167 | 4,174 | 4,180 | 4,169 |
| Incident cases, n | 8 | 17 | 13 | 23 |
| Mean follow-up time (SD), years | 4.86 (1.20) | 4.92 (1.24) | 4.96 (1.23) | 4.89 (1.27) |
| Incidence rate (per 1000 person-years) | 0.39 | 0.83 | 0.63 | 1.13 |
| Rate difference (95%CI), per 1000 person-years | 0.0 (reference) | 0.43 (-0.05, 0.91) | 0.23 (-0.21, 0.67) | 0.73 (0.20, 1.27) |
| Crude HR (95%CI) | 1.0 (reference) | 2.09 (0.90, 4.85) | 1.58 (0.66, 3.82) | 2.85 (1.27, 6.36) |
| Adjusted HR^b^ (95%CI) | 1.0 (reference) | 2.05 (0.88, 4.80) | 1.49 (0.60, 3.71) | 2.57 (1.12, 5.90) |
| **Age≥60 years** |  |  |  |  |
| **Incident VTE** |  |  |  |  |
| Number of participants, n | 1,969 | 1,977 | 1,979 | 1,974 |
| Incident cases, n | 22 | 16 | 28 | 45 |
| Mean follow-up time (SD), years | 4.95 (1.25) | 5.02 (1.31) | 4.95 (1.34) | 4.91 (1.35) |
| Incidence rate (per 1000 person-years) | 2.26 | 1.61 | 2.86 | 4.64 |
| Rate difference (95%CI), per 1000 person-years | 0.0 (reference) | -0.64 (-1.87, 0.59) | 0.61 (-0.81, 2.03) | 2.38 (0.73, 4.03) |
| Crude HR (95%CI) | 1.0 (reference) | 0.71 (0.38, 1.36) | 1.26 (0.72, 2.20) | 2.04 (1.22, 3.39) |
| Adjusted HR^b^ (95%CI) | 1.0 (reference) | 0.64 (0.33, 1.22) | 1.04 (0.59, 1.84) | 1.62 (0.94, 2.80) |
| **Incident PE** |  |  |  |  |
| Number of participants, n | 1,986 | 1,989 | 1,996 | 1,988 |
| Incident cases, n | 12 | 8 | 16 | 23 |
| Mean follow-up time (SD), years | 4.96 (1.23) | 5.03 (1.31) | 4.95 (1.33) | 4.93 (1.34) |
| Incidence rate (per 1000 person-years) | 1.22 | 0.80 | 1.62 | 2.34 |
| Rate difference (95%CI), per 1000 person-years | 0.0 (reference) | -0.42(-1.31, 0.47) | 0.40 (-0.65, 1.45) | 1.13 (-0.05, 2.31) |
| Crude HR (95%CI) | 1.0 (reference) | 0.65 (0.27, 1.60) | 1.32 (0.62, 2.79) | 1.90 (0.95, 3.83) |
| Adjusted HR^b^ (95%CI) | 1.0 (reference) | 0.60 (0.25, 1.47) | 1.16 (0.56, 2.44) | 1.52 (0.67, 3.43) |
| **Incident DVT** |  |  |  |  |
| Number of participants, n | 1,980 | 1,983 | 1,988 | 1,982 |
| Incident cases, n | 10 | 9 | 15 | 23 |
| Mean follow-up time (SD), years | 4.95 (1.25) | 5.02 (1.31) | 4.96 (1.33) | 4.94 (1.34) |
| Incidence rate (per 1000 person-years) | 1.02 | 0.90 | 1.52 | 2.35 |
| Rate difference (95%CI), per 1000 person-years | 0.0 (reference) | -0.11 (-0.97, 0.76) | 0.51 (-0.49, 1.50) | 1.33 (0.18, 2.48) |
| Crude HR (95%CI) | 1.0 (reference) | 0.89 (0.36, 2.19) | 1.49 (0.67, 3.30) | 2.29 (1.09, 4.81) |
| Adjusted HR^b^ (95%CI) | 1.0 (reference) | 0.76 (0.31, 1.87) | 1.09 (0.47, 2.52) | 1.40 (0.62, 3.18) |

HR, hazard ratio; CI, confidence interval; SD, standard deviation; VTE, venous thromboembolism; PE, pulmonary embolism; DVT, deep vein thrombosis; IMFI, intramuscular fat infiltration.

^a^ Q1-Q4 represent quartiles of IMFI in the anterior thigh at baseline. The quartile ranges for each outcome are as follows: For incident VTE: for age<60 years: Q1 (3.17-6.81), Q2 (4.67-7.73), Q3 (5.36-8.93), Q4 (6.21-25.80); for age≥60 years: Q1 (3.50-7.70), Q2 (5.95-8.65), Q3 (6.83-10.12), Q4 (7.97-27.49); For incident PE: for age<60 years: Q1 (3.17-6.81), Q2 (4.67-7.73), Q3 (5.36-8.93), Q4 (6.21-25.80); for age≥60 years: Q1 (3.50-7.70), Q2 (5.96-8.65), Q3 (6.83-10.12), Q4 (7.97-27.49); For incident DVT: for age<60 years: Q1 (3.17-6.81), Q2 (4.67-7.73), Q3 (5.36-8.93), Q4 (6.20-25.80); for age≥60 years: Q1 (3.50-7.70), Q2 (5.95-8.70), Q3 (6.83-10.12), Q4 (7.97-27.49).

^b^ Adjusted for age, sex, BMI, educational attainment, smoking status, alcohol consumption, physical activity levels, white ethnicity, frailty status, weighted CCI, recent surgery, abnormalities of gait and mobility, fracture, sarcopenia, and use of anticoagulant, glucocorticoid, aspirin, and hormone therapy.

**Table S7. Association between IMFI in the anterior thigh and incident** **VTE, PE, and DVT by BMI**

|  | **IMFI in the anterior thigh at baseline** | | | |
| --- | --- | --- | --- | --- |
|  | **Q1 (lowest)**^a^ | **Q2** | **Q3** | **Q4 (highest)** |
| **BMI<30 kg/m^2^** |  |  |  |  |
| **Incident VTE** |  |  |  |  |
| Number of participants, n | 5,002 | 5,015 | 5,026 | 5,011 |
| Incident cases, n | 31 | 32 | 31 | 64 |
| Mean follow-up time (SD), years | 4.88 (1.21) | 4.95 (1.26) | 4.95 (1.26) | 4.93 (1.29) |
| Incidence rate (per 1000 person-years) | 1.27 | 1.29 | 1.25 | 2.59 |
| Rate difference (95%CI), per 1000 person-years | 0.0 (reference) | 0.02 (-0.61, 0.65) | -0.02 (-0.65, 0.61) | 1.32 (0.54, 2.10) |
| Crude HR (95%CI) | 1.0 (reference) | 1.01 (0.62, 1.66) | 0.98 (0.60, 1.61) | 2.03 (1.32, 3.12) |
| Adjusted HR^b^ (95%CI) | 1.0 (reference) | 0.95 (0.58, 1.55) | 0.90 (0.54, 1.48) | 1.75 (1.12, 2.74) |
| **Incident PE** |  |  |  |  |
| Number of participants, n | 5,026 | 5,044 | 5,051 | 5,037 |
| Incident cases, n | 20 | 15 | 15 | 42 |
| Mean follow-up time (SD), years | 4.88 (1.20) | 4.95 (1.25) | 4.95 (1.25) | 4.94 (1.29) |
| Incidence rate (per 1000 person-years) | 0.81 | 0.60 | 0.60 | 1.69 |
| Rate difference (95%CI), per 1000 person-years | 0.0 (reference) | -0.21 (-0.69, 0.26) | -0.21 (-0.68, 0.26) | 0.88 (0.26, 1.50) |
| Crude HR (95%CI) | 1.0 (reference) | 0.74 (0.38, 1.44) | 0.74 (0.38, 1.44) | 2.07 (1.21, 3.53) |
| Adjusted HR^b^ (95%CI) | 1.0 (reference) | 0.70 (0.36, 1.37) | 0.72 (0.36, 1.41) | 1.98 (1.12, 3.50) |
| **Incident DVT** |  |  |  |  |
| Number of participants, n | 5,018 | 5,033 | 5,042 | 5,027 |
| Incident cases, n | 13 | 23 | 18 | 28 |
| Mean follow-up time (SD), years | 4.88 (1.20) | 4.95 (1.26) | 4.95 (1.26) | 4.94 (1.29) |
| Incidence rate (per 1000 person-years) | 0.53 | 0.92 | 0.72 | 1.13 |
| Rate difference (95%CI), per 1000 person-years | 0.0 (reference) | 0.39 (-0.08, 0.87) | 0.19 (-0.25, 0.63) | 0.60 (0.09, 1.10) |
| Crude HR (95%CI) | 1.0 (reference) | 1.74 (0.88, 3.43) | 1.36 (0.67, 2.76) | 2.11 (1.10, 4.08) |
| Adjusted HR^b^ (95%CI) | 1.0 (reference) | 1.60 (0.81, 3.16) | 1.18 (0.58, 2.40) | 1.66 (0.84, 3.28) |
| **BMI≥30 kg/m^2^** |  |  |  |  |
| **Incident VTE** |  |  |  |  |
| Number of participants, n | 1,107 | 1,123 | 1,128 | 1,117 |
| Incident cases, n | 11 | 16 | 20 | 22 |
| Mean follow-up time (SD), years | 4.93 (1.29) | 4.96 (1.29) | 4.91 (1.31) | 4.78 (1.32) |
| Incidence rate (per 1000 person-years) | 2.01 | 2.87 | 3.61 | 4.12 |
| Rate difference (95%CI), per 1000 person-years | 0.0 (reference) | 0.86 (-0.98, 2.70) | 1.60 (-0.38, 3.58) | 2.11 (0.01, 4.21) |
| Crude HR (95%CI) | 1.0 (reference) | 1.42 (0.66, 3.07) | 1.79 (0.86, 3.75) | 2.05 (0.99, 4.23) |
| Adjusted HR^b^ (95%CI) | 1.0 (reference) | 1.37 (0.64, 2.95) | 1.62 (0.76, 3.42) | 1.79 (0.81, 3.98) |
| **Incident PE** |  |  |  |  |
| Number of participants, n | 1,117 | 1,135 | 1,142 | 1,127 |
| Incident cases, n | 6 | 12 | 13 | 11 |
| Mean follow-up time (SD), years | 4.95 (1.27) | 4.98 (1.29) | 4.91 (1.30) | 4.80 (1.30) |
| Incidence rate (per 1000 person-years) | 1.09 | 2.12 | 2.32 | 2.03 |
| Rate difference (95%CI), per 1000 person-years | 0.0 (reference) | 1.04 (-0.44, 2.52) | 1.23 (-0.30, 2.76) | 0.95 (-0.54, 2.43) |
| Crude HR (95%CI) | 1.0 (reference) | 1.95 (0.73, 5.18) | 2.13 (0.81, 5.60) | 1.87 (0.69, 5.06) |
| Adjusted HR^b^ (95%CI) | 1.0 (reference) | 1.87 (0.71, 4.92) | 1.79 (0.67, 4.81) | 1.51 (0.54, 4.24) |
| **Incident DVT** |  |  |  |  |
| Number of participants, n | 1,116 | 1,128 | 1,137 | 1,122 |
| Incident cases, n | 5 | 8 | 11 | 12 |
| Mean follow-up time (SD), years | 4.95 (1.27) | 4.97 (1.28) | 4.92 (1.28) | 4.80 (1.31) |
| Incidence rate (per 1000 person-years) | 0.91 | 1.43 | 1.97 | 2.23 |
| Rate difference (95%CI), per 1000 person-years | 0.0 (reference) | 0.52 (-0.74, 1.79) | 1.06 (-0.35, 2.47) | 1.32 (-0.18, 2.82) |
| Crude HR (95%CI) | 1.0 (reference) | 1.58 (0.52, 4.81) | 2.17 (0.75, 6.25) | 2.45 (0.86, 6.98) |
| Adjusted HR^b^ (95%CI) | 1.0 (reference) | 1.52 (0.49, 4.70) | 2.08 (0.72, 6.04) | 2.27 (0.70, 7.38) |

HR, hazard ratio; CI, confidence interval; SD, standard deviation; VTE, venous thromboembolism; PE, pulmonary embolism; DVT, deep vein thrombosis; IMFI, intramuscular fat infiltration.

^a^ Q1-Q4 represent quartiles of IMFI in the anterior thigh at baseline. The quartile ranges for each outcome are as follows: For incident VTE: for BMI<30 kg/m^2^: Q1 (3.17-7.65), Q2 (4.63-8.35), Q3 (5.24–9.65), Q4 (5.97-26.40); for BMI≥30 kg/m^2^: Q1 (3.66-9.28), Q2 (5.66-10.26), Q3 (6.31-11.76), Q4 (7.34-27.49); For incident PE: for BMI<30 kg/m^2^: Q1 (3.17-7.65), Q2 (4.63-8.35), Q3 (5.24-9.69), Q4 (5.97-26.40); for BMI≥30 kg/m^2^: Q1 (3.66-9.35), Q2 (5.66-10.36), Q3 (6.31-12.01), Q4 (7.34-27.49); For incident DVT: for BMI<30 kg/m^2^: Q1 (3.17-7.65), Q2 (4.62-8.35), Q3 (5.24-9.69), Q4 (5.96-26.40); for BMI≥30 kg/m^2^: Q1 (3.66-9.29), Q2 (5.66-10.26), Q3 (6.31-11.76), Q4 (7.34-27.49).

^b^ Adjusted for age, sex, BMI, educational attainment, smoking status, alcohol consumption, physical activity levels, white ethnicity, frailty status, weighted CCI, recent surgery, abnormalities of gait and mobility, fracture, sarcopenia, and use of anticoagulant, glucocorticoid, aspirin, and hormone therapy.
